# The recurrence rate of ovarian endometrioma in women aged 40–49 years and impact of hormonal treatment after conservative surgery

**DOI:** 10.1038/s41598-020-73434-0

**Published:** 2020-10-05

**Authors:** Nara Lee, Seunggi Min, Seyeon Won, Yeon Jean Cho, Miseon Kim, Mi Kyoung Kim, Yong Wook Jung, Bo Seong Yun, Seok Ju Seong, Mi-La Kim

**Affiliations:** 1 Department of Obstetrics and Gynecology, CHA Gangnam Medical Center, CHA University School of Medicine, 566, Nonhyeon-ro, Gangnam-gu, Seoul, 06135 Republic of Korea; 2 Department of Obstetrics and Gynecology, Dong-A University Medical Center, Dong-A University College of Medicine, Busan, Republic of Korea

**Keywords:** Diseases, Risk factors

## Abstract

The aim of this study was to evaluate the rate of and risk factors for recurrence ovarian endometrioma after conservative surgery in patients aged 40–49 years. This retrospective, single-center study included 408 women between January 2008 and November 2018. All patients underwent ovarian cyst enucleation, were pathologically diagnosed with ovarian endometrioma and were followed up for ≥ 6 months. Recurrence was defined as a cystic mass with diameter ≥ 2 cm detected by sonography. Recurrence rate after conservative surgery and risk factor of recurrence were analyzed. The median follow-up duration after surgery was 32.0 ± 25.9 months (range 6–125 months). Ovarian endometrioma recurred in 34 (8.3%) of included women and median time to recurrence was 22.4 ± 18.2 months. The cumulative recurrences rate at 12, 24, 36, and 60 months were 3.7%, 6.7%, 11.1%, and 16.7%, respectively. Recurrence was correlated with multilocular cysts (*p* = 0.038), previous surgical history of ovarian endometrioma (*p* = 0.006) and salpingectomy (*p* = 0.043), but not use or duration of post-operative medication. In multivariate analysis, large cyst size (> 5.5 cm) was only risk factor for recurrence in this age group. Post-operative medication did not reduce disease recurrence rate, and thus may be administered for endometriosis-associated pain rather than to prevent recurrence in patients aged 40–49 years.

## Introduction

Ovarian endometrioma is one of the most common benign gynecologic diseases with an estimated incidence of 10–20% in women of reproductive age^1^. Ovarian endometrioma is most prevalent in women between 40 and 44 years, and the incidence of this disease tends to decrease after menopause^2^.

Conservative surgery is the standard treatment for ovarian endometrioma. However, the recurrence rate after conservative laparoscopic surgery is high^3^, having been reported to be 21.5% at 2 years post-surgical and 40–50% at 5 years post-surgical among all age groups^4^. Moreover, after second-line conservative laparoscopic surgery, cumulative recurrence rates were reported to be 13.7% at 2 years post-surgical and 37.5% at 5 years post-surgical^5^. Therefore, additional medical treatments are recommended in addition to conservative surgery to prevent or delay the recurrence of ovarian endometrioma.

In 2014, the European Society of Human Reproduction and Embryology (ESHRE) recommended ovarian cystectomy rather than drainage and coagulation of endometriosis in cases of surgical treatment, since ovarian cystectomy can effectively reduce endometriosis-associated pain and has a lower disease recurrence rate^6,7^. Since the two primary symptoms of endometriosis are pain and infertility, ESHRE guidelines recommend the use of post-operative hormonal therapy for at least 18 to 24 months for secondary disease prevention in women not immediately seeking conception, as well as for the prevention of endometriosis-associated dysmenorrhea^8^.

However, there are limitations to post-operative medication in older women (40–49 years). Various medical conditions such as hypertension, diabetes mellitus, thromboembolic disease, cardiovascular problems, and osteoporosis are more frequent in this group than in younger women. Moreover, no age-based drug administration guidelines exist, and few studies have investigated the recurrence rate of ovarian endometrioma in women aged 40–49 years or differences in recurrence rates according to post-operative medication^9,10^.

The purpose of this study was to investigate the recurrence rates of ovarian endometrioma and the factors affecting recurrence in women aged 40–49 years. We also aimed to determine whether post-operative hormonal medication reduces disease recurrence in this age group.

## Methods

### Patient population and data collection

All processes conducted in this study involving human participants were in accordance with the ethical standards of the institutional committee and with the 1964 Helsinki declaration and its later amendments or comparable ethical standards. We retrospectively reviewed data from patients aged 40–49 years and treated with conservative surgery for ovarian endometrioma from January 2008 to November 2019. The study protocol was approved by the Institutional Review Board on the CHA Gangnam Medical Center (GCI-19–36). Institutional Review Board on the CHA Gangnam Medical Center waived the need for informed consent as part of their study approval. In this study, total 22 surgeons were included. All of them were experienced surgeons with more than 300 cases of experience of laparoscopic surgeries and more than 100 cases of laparotomies. The surgical procedure was as follows: first, exfoliated around the endometrioma to enable mobilization. A sharp cortical incision was made on the anti-mesenteric border, and a cleavage plane was identified by sharp- or hydro-dissection. After the entire cyst was removed, the ovarian bed and additional endometriosis of peritoneum and ovarian fossa was fulgurated by bipolar electrocautery.

Patients were included in the study based on the following inclusion criteria: (1) pathologically confirmed ovarian endometriosis; (2) followed up at least six months after surgery; and (3) ultrasonography performed in order to determine recurrence of ovarian endometrioma after surgery. All patients were offered clinical follow-up at intervals ranging from 3 to 12 months or when medical evaluation was needed. At every follow-up visit, a transvaginal/transrectal sonography (TVS/TRS) examination was performed, and symptoms, medical treatment, and clinical data were recorded.

The exclusion criteria were as follows: (1) patients who underwent oophorectomy; (2) patients who were diagnosed with associated gynecologic malignancy; (3) patients with revised American Society for Reproductive Medicine (rASRM) classification I or II; (4) patients with subsequent pregnancy after surgery; or 5) menopausal status at the time of surgery.

**Figure 1:**
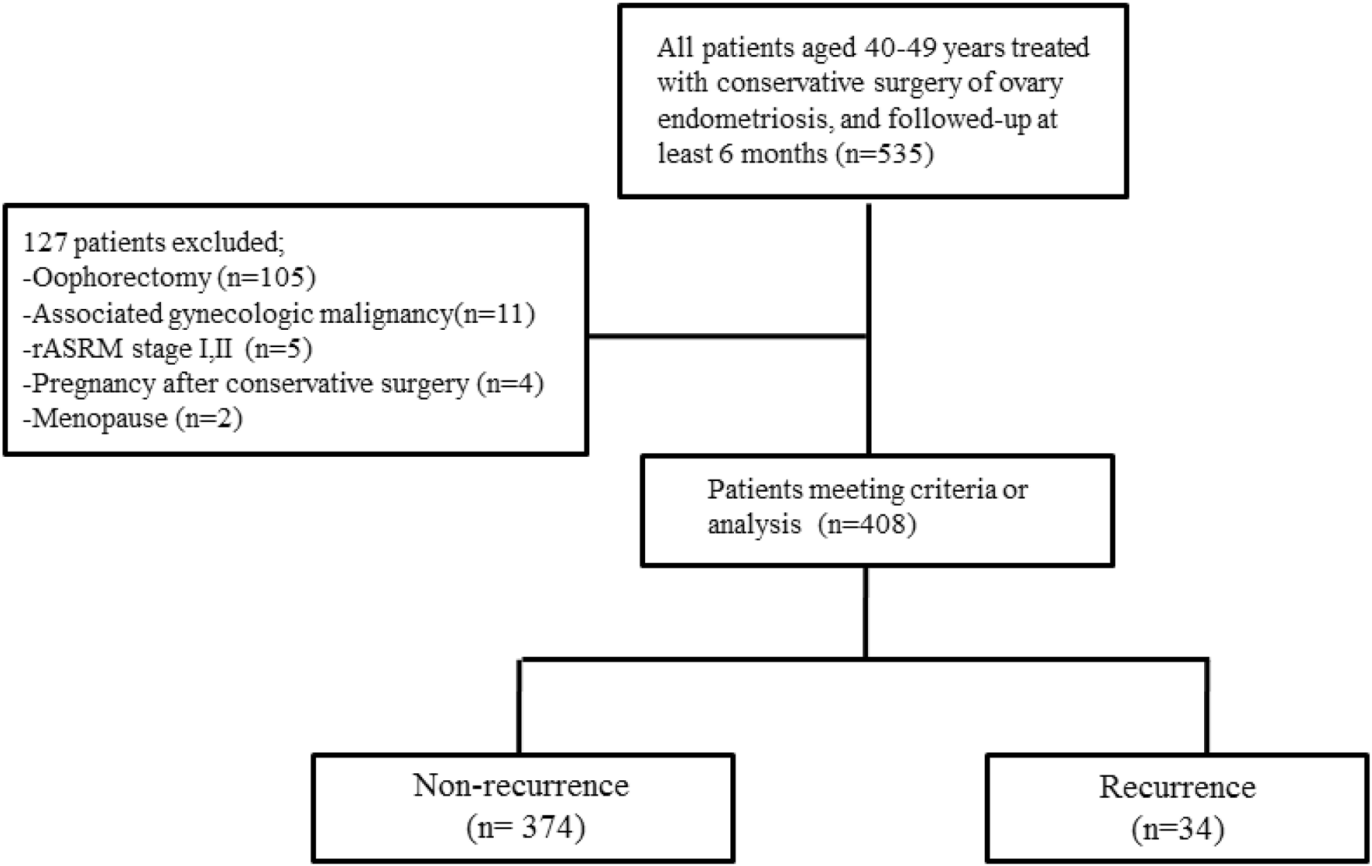
Flow diagram of patient selection process.

Initially, 535 patients were screened. Among them, 127 patients were excluded based on the above criteria, and a total of 408 patients were selected for this study (Fig. 1). The medical charts of all patients were reviewed to collect data on demographics, age at surgery, body mass index, gravidity, parity, serum cancer antigen 125 (CA125) levels, size of the endometrioma, rASRM stage, use of post-operative medications, and time to disease recurrence.

### Definition of recurrence

Recurrence of ovarian endometrioma was defined when TVS or TRS indicated the presence of a cystic mass with a minimum diameter of 20 mm, thick walls, irregular margins, homogenous low echogenic fluid content with scattered internal echoes, and the absence of papillary proliferations^11^. If a patient had two endometriomas that were < 20 mm, and the sum of their diameters was > 20 mm, the patient was considered to have recurrent ovarian endometrioma.

### Statistical analysis

Statistical analysis was performed using SPSS 25.0 software (SPSS Chicago, IL, USA). The Chi-square and Fisher’s exact tests were used for analysis of categorical variables. Quantitative variables were compared using the Mann–Whitney U test. The Kaplan–Meier method was used to calculate the cumulative probability of recurrence, and the comparison between the curves was performed using the log-rank test. Multivariate modeling using Cox’s proportional hazards models, including the significant variables in univariate analysis, were used to obtain a subset of independent risk factors of recurrent ovarian endometrioma, and among these variables, those with *p* value < 0.2 underwent multivariate regression analyses. A *p* value < 0.05 was considered statistically significant.

### Ethics approval

The study protocol was approved by the Institutional Review Board on the CHA Gangnam Medical Center (GCI-19-36).

### Informed consent

Informed consent was not sought as a retrospective study design was used. Data were anonymized and de-identified before analysis, and therefore, informed consent was not required.

## Results

### Patient characteristics

A total of 408 patients were included in this study. The median follow-up duration after surgery was 32.0 months (range 6–125 months). The baseline clinical and surgical characteristics of the patients are shown in Table 1. At the time of conservative surgery, the mean age of the patients was 42.7 years. Forty-seven (11.5%) patients had a history of previous surgery for ovarian endometrioma. Of the 408 patients, 339 (83.1%) received post-operative medication on the basis of individual characteristics, and 172 (42.2%) patients were treated with post-operative medication for more than 12 months. The median duration of medication was 15.4 ± 17.3 months (range 0–111 months).

**Table 1:** Baseline characteristics of patients with 40–49 years (n = 408).

Characteristics	Mean ± SD (range), or n(%)
Age (years)	42.7 ± 2.3 (42, 40–49)
Gravidity	1.6 ± 1.4 (2, 0–6)
Parity	1.1 ± 0.9 (1, 0–3)
Nulliparity	150 (36.8%)
Weight (kg)	57.5 ± 8.5 (56, 40–92)
Height (cm)	160.7 ± 5.1 (160, 146.8–175.5)
BMI (kg/m^2^)	22.3 ± 3.1 (21.5, 15.3–36.7)
Tumor size (cm)	5.9 ± 3.0 (5.5, 0.9–15.4)
**Cyst character**	
Unilocular	260 (63.7%)
Multilocular	148 (36.3%)
CA125 (U/mL) (n = 349)	71.4 ± 82.9 (48.1,7.26–1017)
AMH (ng/mL) (n = 183)	0.96 ± 1.12 (0.56, 0.03–8.02)
**rASRM Stage**	
III	175 (42.9%)
IV	233 (57.1%)
**Main symptom**	
Pain	230 (56.4%)
Compression symptom	6 (1.5%)
Bleeding	46 (11.3%)
Infertility	5 (1.2%)
Growing ovarian cyst	25 (6.1%)
Incidentally detected	96 (23.5%)
**Laterality**	
Unilateral	293 (71.8%)
Bilateral	115 (28.2%)
**Cul-de-sac obliteration**	
None	107 (26.2%)
Obliterated	301 (73.8%)
**Previous surgical history of ovarian endometrioma**	
No	361 (88.5%)
Yes	47 (11.5%)
**Extent of surgery: uterus**	
Uterus preservation	327 (80.1%)
Hysterectomy	81 (19.9%)
**Extent of surgery: tube**	
No	319 (78.2%)
One tube	33 (8.1%)
Both tubes	56 (13.7%)
**Associated myoma or adenomyosis**	
No	79 (19.4%)
Yes	329 (80.6%)
**Surgical method**	
Laparoscopy or robotic surgery	377 (92.4%)
Explo-laparotomy	31 (7.6%)
**Postoperative medical treatment**	
No	69 (16.9%)
Yes	339 (83.1%)
**Duration of medical treatment**	
No medication	69 (16.9%)
> 0 ~ ≤ 6Mo	74 (18.1%)
> 6 ~ ≤ 12Mo	93 (22.8%)
> 12 ~ ≤ 24Mo	106 (26.0%)
> 24 ~ ≤ 36Mo	26 (6.4%)
> 36Mo	40 (9.8%)
Median time of medical treatment (month)	15.4 ± 17.3 (11, 0–111)
**Recurrent ovarian endometrioma (≥ 2 cm)**	
No	374 (91.7%)
Yes	34 (8.3%)
Follow-up duration (month)	32.0 ± 25.9 (23, 6–125)
Median time to recurrence(month) (n = 34)	22.4 ± 18.2 (19.5, 3–76)
Median time to reoperation in recurrent case (month) (n = 11)	34.0 ± 14.0 (37, 12–55)

BMI, body mass index; CA125, cancer antigen 125; AMH, anti-Mullerian hormone; rASRM, revised American Society for Reproductive Medicine; Mo, month.

The type and duration of post-operative medication was chosen on the basis of individual characteristics The most commonly used post-operative medication was oral progestin in 223 patients (54.7%): oral progestin only in 124 patients and combined with other medications in 99 patients. GnRH agonist was used in 145 patients (35.5%): GnRH only in 38 (9.3%) and combined with other medication in 107 patients (Table 2).

**Table 2:** Types of post-operative medications (n = 408).

Types of post-operative medications	Number (%)
GnRH agonist	38 (9.3%)
GnRH agonist + oral progestin	41 (10.0%)
GnRH agonist + oral progestin + OC	8 (2.0%)
GnRH agonist + oral progestin + OC + progestin IUD	1 (0.2%)
GnRH agonist + oral progestin + progestin IUD	8 (2.0%)
GnRH agonist + OC	22 (5.4%)
GnRH agonist + OC + progestin IUD	2 (0.5%)
GnRH agonist + progestin IUD	25 (6.1%)
Oral progestin	124 (30.4%)
Oral progestin + OC	23 (5.6%)
Oral progestin + progestin IUD	18 (4.4%)
OC	10 (2.5%)
OC + progestin IUD	3 (0.7%)
Progestin IUD	16 (3.9%)
No medication	69 (16.9%)
GnRH (multiple choices)	145 (35.5%)
Oral progestin (multiple choices)	223 (54.7%)
OC (multiple choices)	68 (16.7%)
Progestin IUD (multiple choices)	72 (17.6%)
No medication	69 (16.9%)

GnRH agonist, gonadotrophin releasing hormone agonist; OC, oral contraceptives; IUD, intrauterine device.

### Recurrence rate and characteristics of recurrence cases

**Figure 2:**
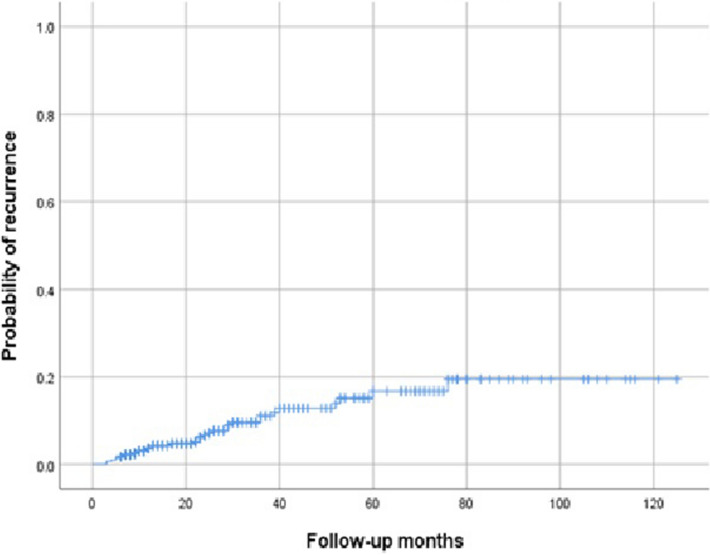
Cumulative recurrence of endometrioma after conservative ovarian cyst enucleation in women with 40–49 years.

**Figure 3:**
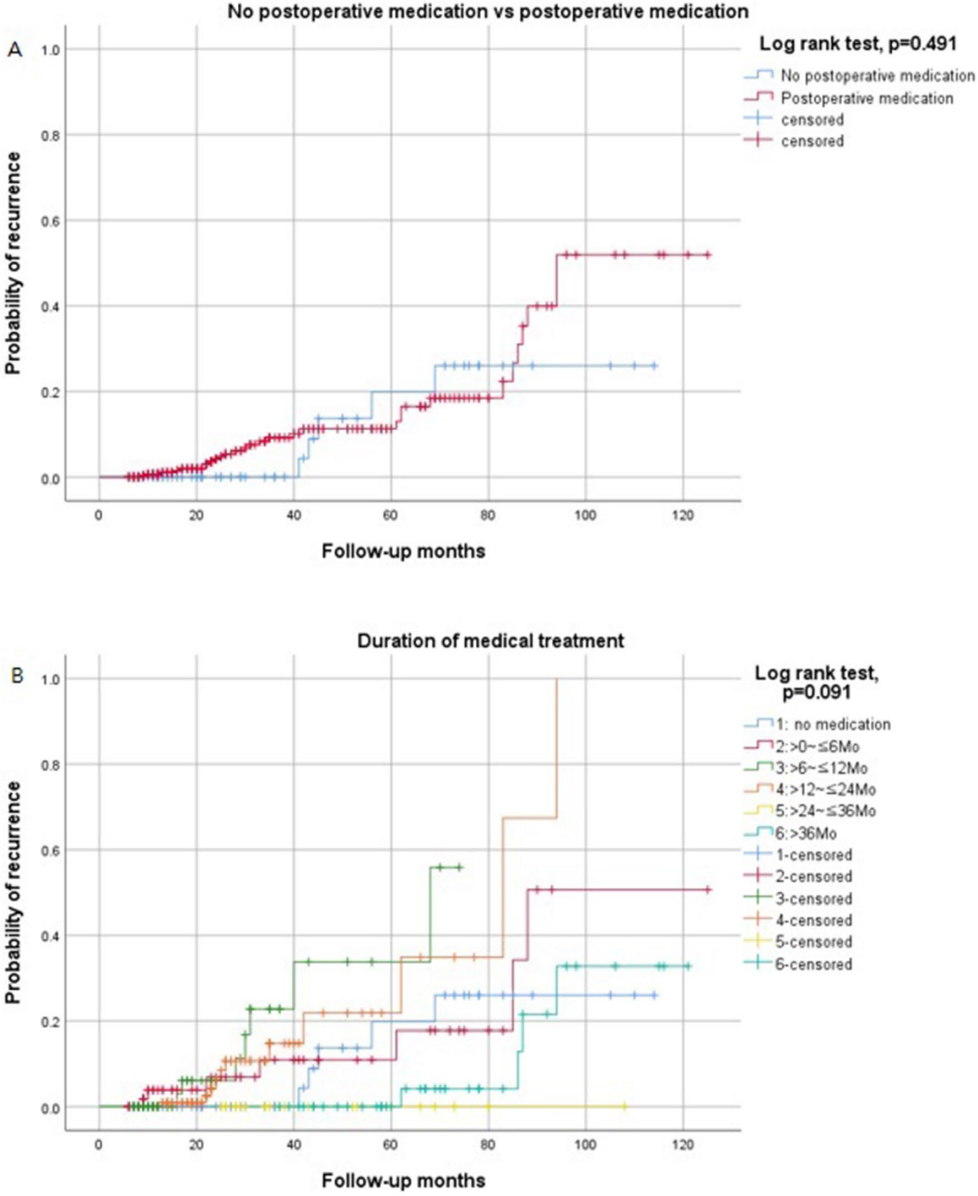
Probability of recurrence by Kaplan- Meire curve (Log-rank test) (**A**). no medication vs. postoperative medication (**B**). Duration of medican treatment.

Following the given definition of recurrence, 34 (8.3%) patients experienced recurrent ovarian endometrioma. Excluding one of 34 patients who experienced recurrent endometrioma, follow-up was performed, and 11 (2.7%) patients underwent subsequent surgical intervention. The baseline patient characteristics of the recurrence cases are shown in Table 3. The median time to recurrence was 22.4 months (range 3–76 months). The cumulative recurrence rates at 12, 24, 36, and 60 months after conservative surgery were 3.7%, 6.7%, 11.1%, and 16.7%, respectively (Fig. 2). There were statistically significant differences between the recurrence group and the non-recurrence group in tumor size (7.3 ± 3.7 versus 5.8 ± 2.9 cm, *p* = 0.041), cyst characteristics (unilocular versus multilocular, *p* = 0.041), initial CA125 level (99.3 ± 79.4 versus 68.8 ± 82.9 U/mL, *p* = 0.037), and history of previous surgery for ovarian endometrioma (p = 0.009). However, there were no statistically significant differences between the two groups in the use of post-operative medication or the duration of medical treatment (Table 3). The Kaplan–Meier curve also showed no statistically significant differences in the use of post-operative medication (Fig. 3A) and duration of medical treatment (Fig. 3B) by log-rank test.

**Table 3:** Analysis of possible risk factors for recurrent ovarian endometrioma (n = 408).

Covariable	Nonrecurrent (n = 374)	Recurrent (n = 34)	p-value
Age (years)^a^	42.7 ± 2.4 (42, 40–49)	42.3 ± 2.0 (42, 40–47)	0.481
**Age group**^**b**^			0.508
40–44	297 (79.4%)	29 (85.3%)	
45–49	77 (20.6%)	5 (14.7%)	
Gravidity^a^	1.5 ± 1.4 (2, 0–6)	1.6 ± 2.0 (2, 0–6)	0.877
Parity^a^	1.1 ± 1.0 (1, 0–3)	1.0 ± 0.9 (1, 0–3)	0.504
Nulliparity^b^	138 (36.9%)	12 (35.3%)	1.000
Weight (kg)^a^	57.5 ± 8.4 (56.1, 40–87.6)	58.1 ± 9.8 (55.6, 46–92)	0.960
Height(cm)^a^	160.6 ± 5.1 (160, 146.8–175.5)	162.1 ± 5.6 (160.2, 154–175)	0.089
BMI (kg/m^2^)^a^	22.3 ± 3.1 (21.6, 15.3–36.7)	22.1 ± 3.4 (21.0, 18.6–32.1)	0.349
Tumor size (cm)^a^	5.8 ± 2.9 (5.4, 0.9–15.3)	7.3 ± 3.7 (6.3, 2.0–15.4)	0.041
**Cyst character**^**b**^			0.041
Unilocular	244 (65.2%)	16 (47.1%)	
Multilocular	130 (34.8%)	18 (52.9%)	
CA125(U/mL) (n = 349)^a^	68.8 ± 82.9 (46.4, 7.26–1017.0) (n = 321)	99.3 ± 79.4 (72.5, 10.1–281.7) (n = 29)	0.037
AMH (ng/mL) (n = 183)^a^	0.92 ± 1.12 (0.54, 0.03–8.02) (n = 164)	1.24 ± 1.12 (0.85, 0.06–3.49) (n = 19)	0.170
**rASRM stage**^**b**^			0.106
III	165 (44.1%)	10 (29.4%)	
IV	209 (55.9%)	24 (70.6%)	
**Main symptoms**^**c**^			0.500
Pain	209 (55.9%)	21 (61.8%)	
Compression symptom	6 (1.6%)	0 (0%)	
Bleeding	42 (11.2%)	4 (11.8%)	
Infertility	5 (1.3%)	0 (0%)	
Growing ovarian cyst	21 (5.6%)	4 (11.8%)	
Incidentally detected	91 (24.3%)	5 (14.7%)	
**Laterality**^**b**^			0.327
Unilateral	271 (72.5%)	22 (64.7%)	
Bilateral	103 (27.5%)	12 (35.3%)	
**Cul-de-sac obliteration**^**b**^			0.222
None	102 (27.3%)	5 (14.7%)	
Partial	91 (24.3%)	8 (23.5%)	
Complete	181 (48.4%)	21 (61.8%)	
**Previous surgical history of ovarian endometrioma**^**b**^			0.009
No	336 (89.8%)	25 (73.5%)	
Yes	38 (10.2%)	9 (26.5%)	
**Extent of surgery: Uterus**^**b**^			1.000
Uterus preservation	300 (80.2%)	27 (79.4%)	
Hysterectomy	74 (19.8%)	7 (20.6%)	
**Extent of surgery: Tube**^**c**^			0.286
No	293 (78.3%)	26 (76.5%)	
One tube	32 (8.6%)	1 (2.9%)	
Both tube	49 (13.1%)	7 (20.6%)	
**Associated myoma or adenomyosis**^**b**^			1.000
No	73 (19.5%)	6 (17.6%)	
Yes	301 (80.5%)	28 (82.4%)	
**Surgical method**^**b**^			0.312
Laparoscopy or Robotic	347 (92.8%)	30 (88.2%)	
Explo-laparotomy	27 (7.2%)	4 (11.8%)	
**Postoperative medical treatment**^**b**^			0.628
No	62 (16.6%)	4 (11.8%)	
Yes	312 (83.4%)	30 (88.2%)	
**Duration of medical treatment**^**c**^			0.639
No medication	64 (17.1%)	5 (14.7%)	
> 0 ~ ≤ 6Mo	67 (17.9%)	7 (20.6%)	
> 6 ~ ≤ 12Mo	86 (23.0%)	7 (20.6%)	
> 12 ~ ≤ 24Mo	95 (25.4%)	11 (32.3%)	
>24~ ≤ 36 Mo	26 (7.0%)	0 (0%)	
> 36 Mo	36 (9.6%)	4 (11.8%)	
Median time of medical treatment (month)^a^	15.4 ± 17.3 (11, 0–111)	16.3 ± 17.9 (12, 0–66)	0.815

BMI, body mass index;CA125, cancer antigen 125; AMH, anti-Mullerian hormone; rASRM, revised American Society for Reproductive Medicine; Mo, month.

^a^Mann-Whitney U test.

^b^Fisher’s exact test.

^c^Chi-square test.

**Figure 4:**
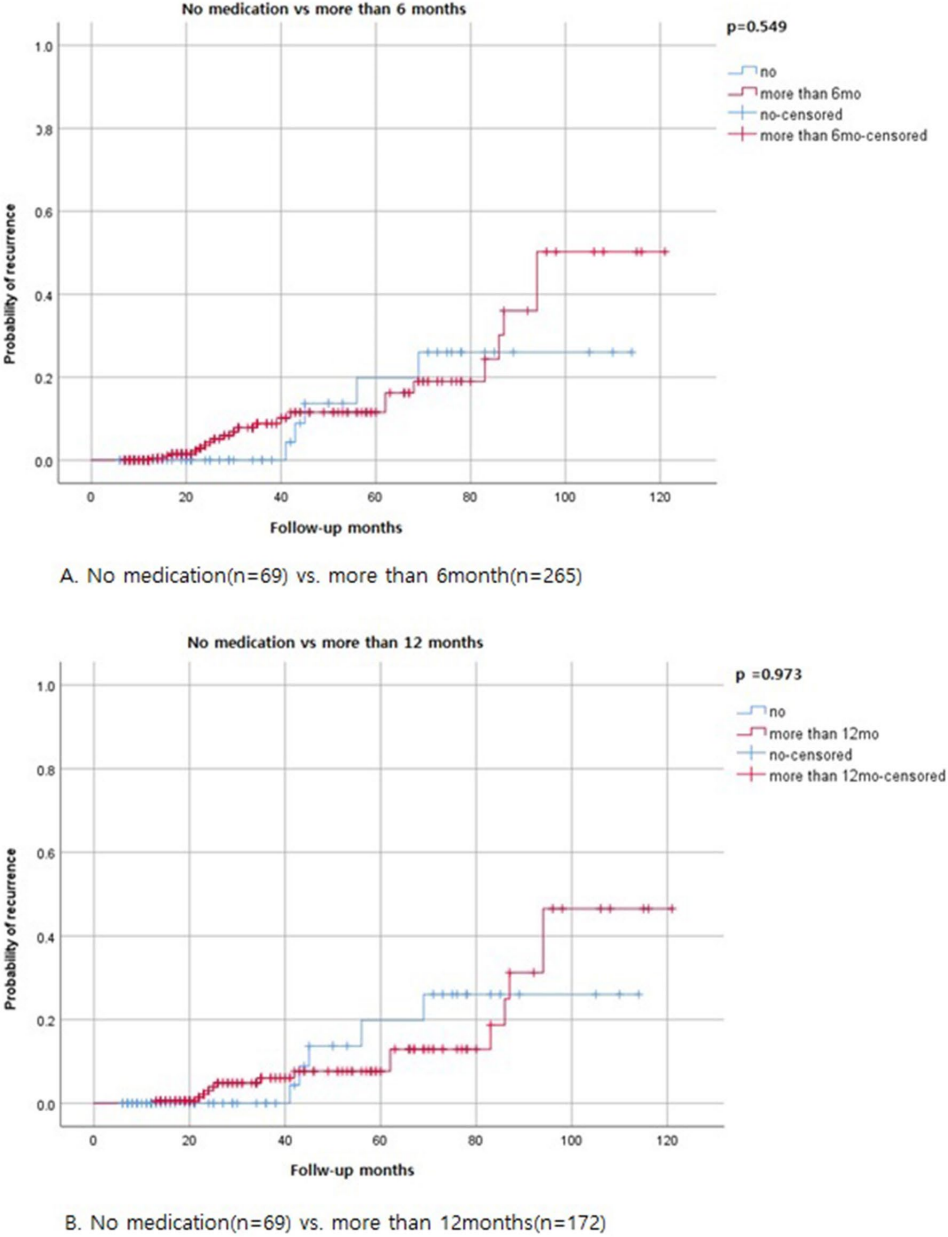

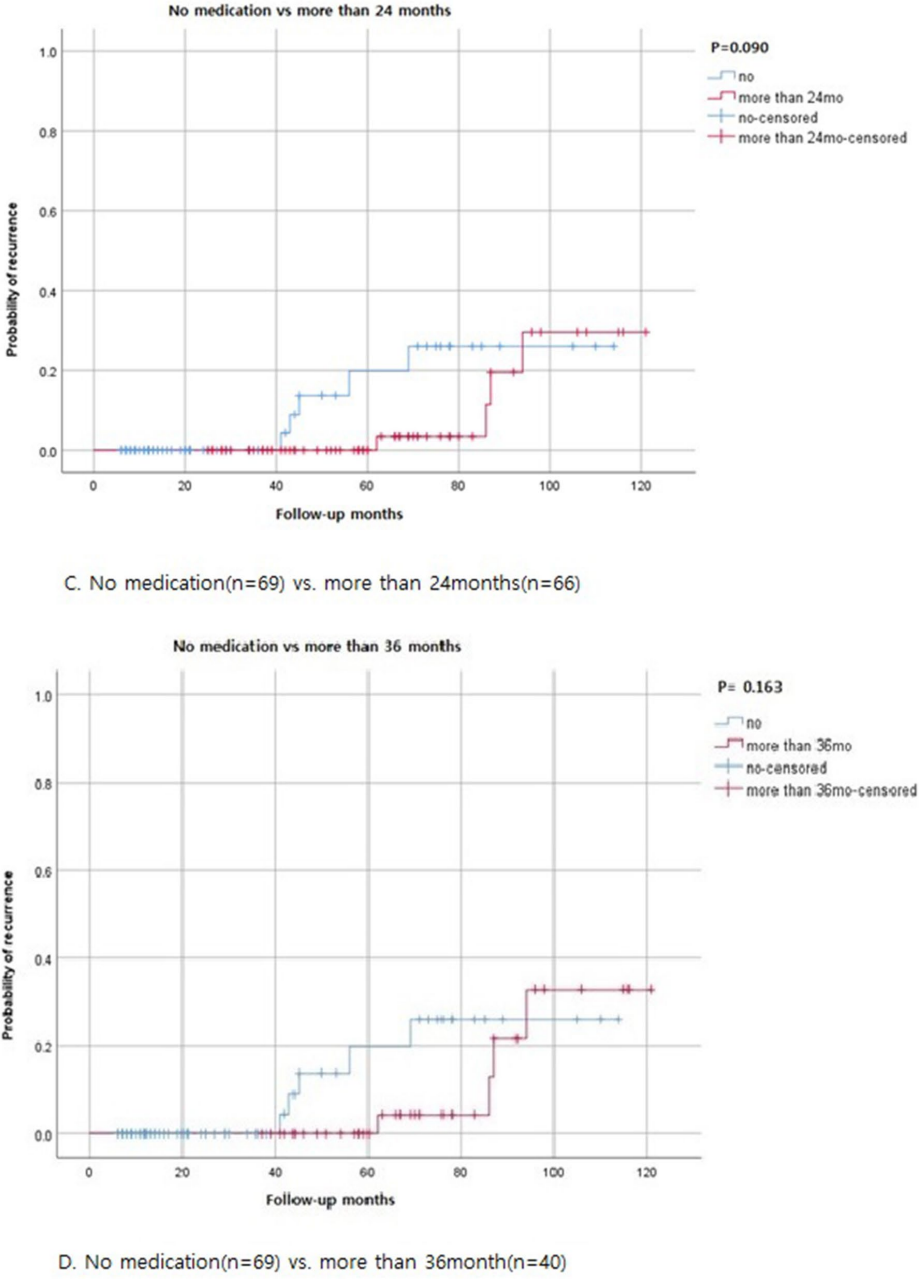
Subgroup analysis of recurrence rates in no medication vs duration of medications. (Log-rank test).

In this study, sub-analysis was performed according to the duration of hormonal therapy after conservative surgery (Fig. 4) In subgroup analysis, there are no significant differences of recurrence rate. (no medication group vs. more than 6mo (months) medication group; *p* = 0.549, no medication group vs. more than 12mo medication group; *p* = 0.973, no medication group vs. more than 24mo medication group; *p* = 0.090, no medication group vs. more than 36mo medication group; *p* = 0.163).

### Univariate and multivariate analysis for recurrence

**Figure 5:**
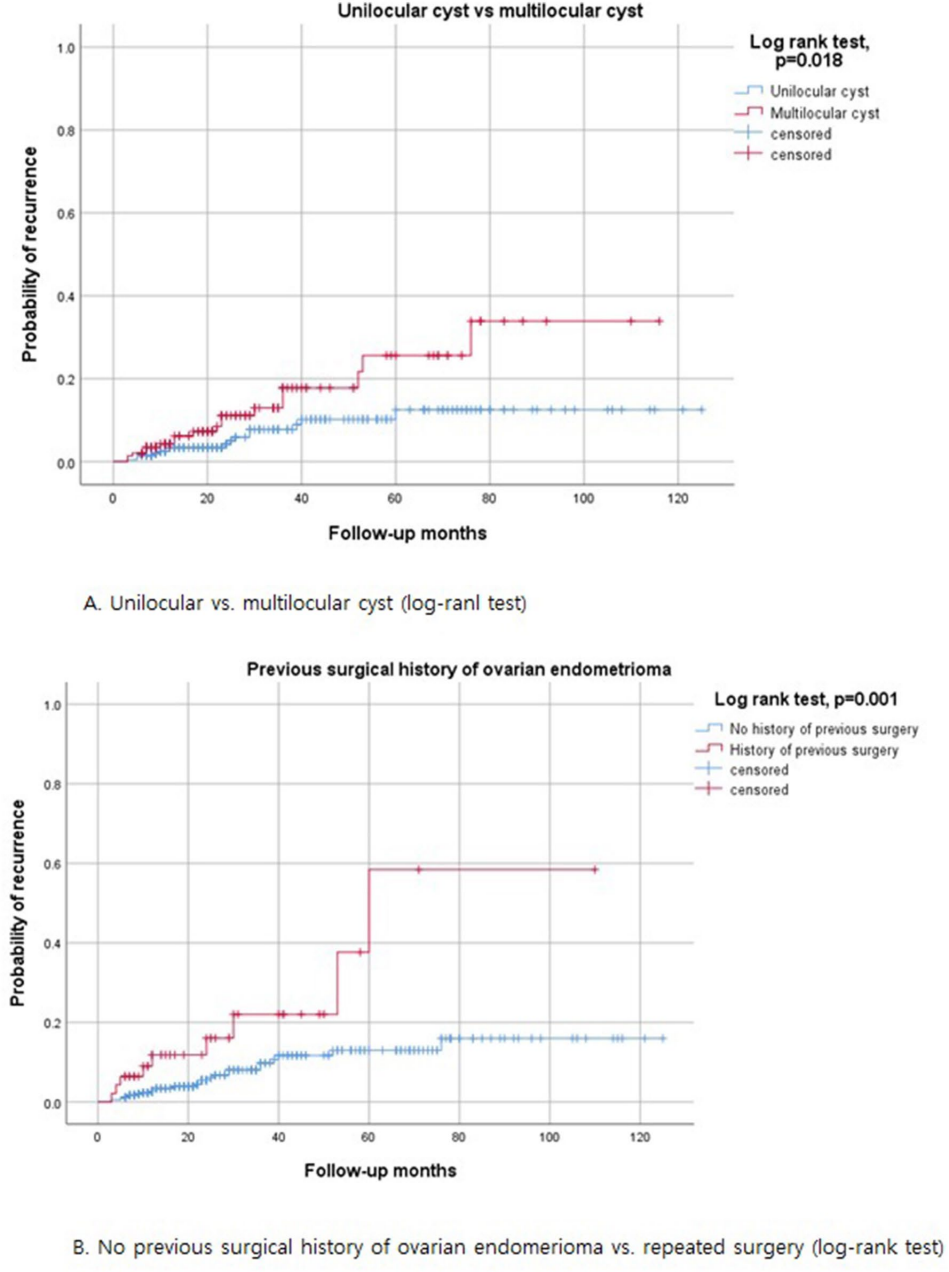

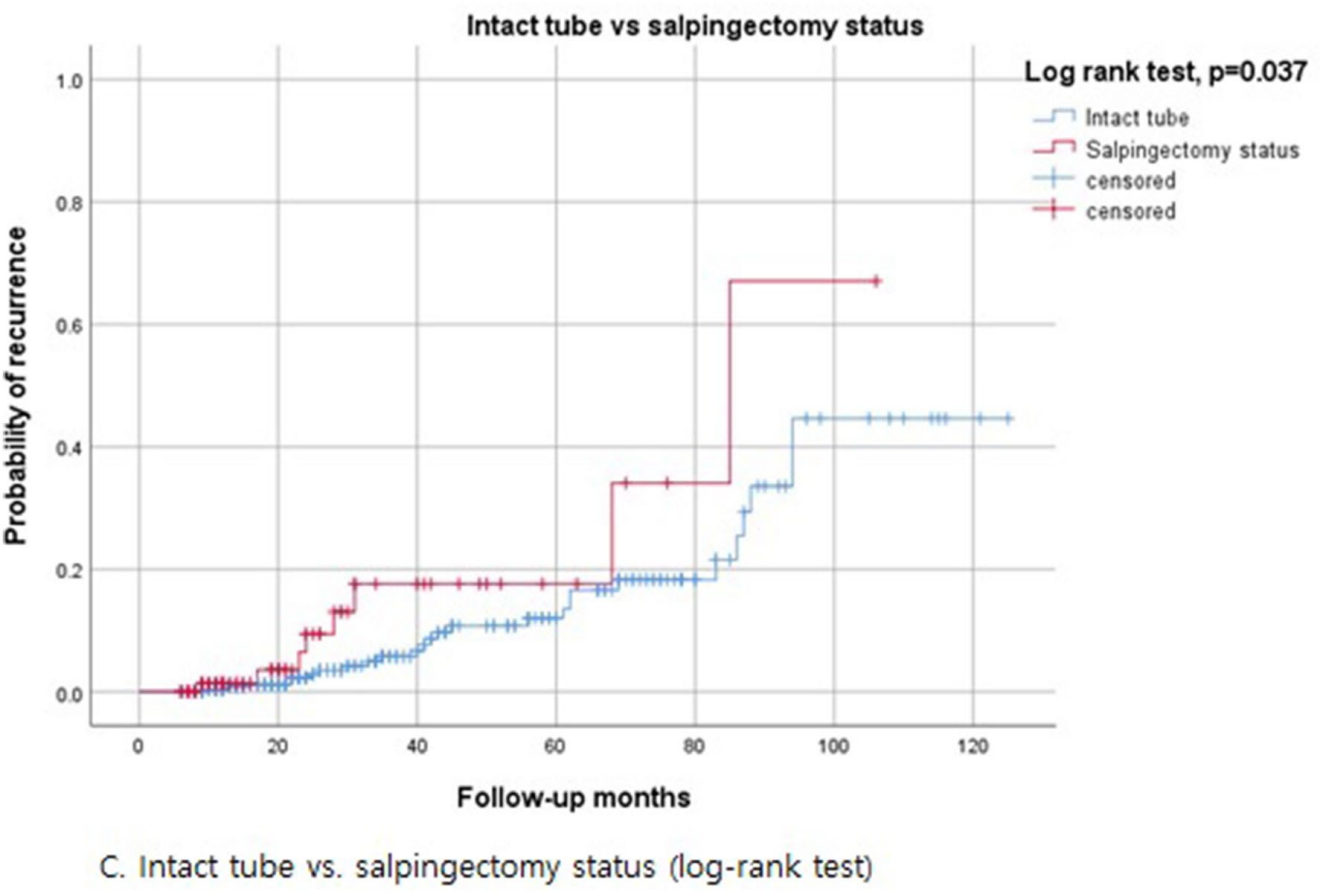
Kaplan-Meire curve of 3 possible risk factors for recurrent endometrioma on univariate analysis.(Log-rank test).

Cox regression analysis was performed on the univariate and multivariate analysis of risk factor for post-operative recurrence of ovarian endometriosis in patients 40–49 years of age (Table 4). According to the univariate analysis, recurrence was correlated with multilocular cysts (*p* = 0.030) and a previous surgical history of ovarian endometrioma (*p* = 0.001), and salpingectomy (*p* = 0.043). Log-rank tests also showed statistical significance with a higher recurrence rate in patients with multilocular cysts (*p* = 0.018) and a previous surgical history of ovarian endometrioma (*p* = 0.001), and salpingectomy (*p* = 0.037) (Fig. 5). The recurrence rate was not correlated with tumor size, initial CA125 level, post-operative medication, or duration of post-operative medication. Using multivariate analysis by Cox regression analysis, tumor size (> 5.5 cm) was only risk factor for disease recurrence in patients aged 40–49 years (*p* = 0.040).

**Table 4:** Univariate and multivariate analysis for independent risk factors of recurrent ovarian endometrioma by Cox proportional hazards models (n = 408).

Characteristics	Univariate		Multivariate	
Risk factors of recurrence	HR (95% CI)	p-value	HR (95% CI)	p-value
Age > 42 years	1.204 (0.600–2.415)	0.601		
Gravidity > 2	0.901 (0.392–2.073)	0.806		
Parity > 1	0.501 (0.239–1.053)	0.068	0.574 (0.248–1.325)	0.193
Weight > 56 kg	0.925 (0.466–1.834)	0.823		
Height > 160.7 cm	1.030 (0.525–2.021)	0.930		
BMI > 21.5 kg/m^2^	0.811 (0.409–1.609)	0.549		
Tumor size > 5.5 cm	1.958 (0.967–3.965)	0.062	2.462 (1.040–5.830)	0.040
Multilocular (vs unilocular)	2.110 (1.074–4.143)	0.030	1.349 (0.602–3.026)	0.468
CA125 > 48.1U/mL (n = 349)	2.019 (0.950–4.293)	0.068	1.521 (0.640–3.612)	0.342
AMH > 0.56 ng/mL (n = 183)	0.974 (0.368–2.576)	0.957		
rASRM stage IV (vs III)	1.742 (0.830–3.657)	0.142	0.740 (0.247–2.219)	0.591
Pain symptom (vs no pain symptom)	0.605 (0.302–1.215)	0.509		
Bilateral (vs unilateral)	1.491 (0.736–3.018)	0.267		
CDS obliteration (vs no)	1.929 (0.743–5.004)	0.177	2.014 (0.492–8.248)	0.330
Incomplete surgery (vs complete)	0.586 (0.264–1.299)	0.188	0.559 (0.230–1.356)	0.198
Previous surgical history of ovarian endometrioma (vs no)	3.890 (1.805–8.385)	0.001	2.378 (0.896–6.313)	0.082
Hysterectomy (vs uterus preservation)	1.581 (0.686–3.644)	0.282		
Salpingectomy (vs no)	2.294 (1.027–5.125)	0.043	2.008 (0.686–5.873)	0.203
Associated myoma/adenomyosis (vs no)	1.423 (0.583–3.472)	0.439		
Explo-laparotomy (vs laparoscopic or robotic)	0.819 (0.281–2.388)	0.715		
Postoperative medication (vs no)	1.393 (0.538–3.603)	0.495		
Duration of medication > 11Mo (vs ≤ 11Mo)	0.782 (0.398–1.536)	0.475		

HR, hazard ratio; CI, confidence interval; BMI, body mass index; CA125, cancer antigen 125; AMH, anti-Mullerian hormone; rASRM, revised American Society for Reproductive Medicine; CDS, cul-de-sac; d/t, due to; endo, endometriosis; Mo, month.

## Discussion

The aim of the current study was to evaluate the recurrence of ovarian endometrioma in patients aged 40–49 years and to analyze the risk factors for recurrence, especially the use of post-operative medication, in this age group. The cumulative recurrence rates of ovarian endometrioma reported herein were relatively low compared to the meta-analysis by Guo, which showed recurrence rates of 21.5% and 40–50% at 24 and 60 months after conservative surgery, respectively^4^. In our study, the only risk factor for recurrence of ovarian endometrioma in this age group was large cyst size (cyst size > 5.5 cm). Two previous studies reported the recurrence rates of ovarian endometrioma in women aged 40–49 years. He et al. reported that the cumulative recurrence rate was 10.0% at 1 year and 27% at 5 years after surgery in patients aged 45 years higher, and that the risk of recurrence was reduced with post-operative treatment and increased by ovarian preservation^9^. However, this study included oophorectomized patients and used a different definition of recurrence, as not only sonographic findings but also recurrent pain symptoms and elevated serum CA125 level were considered as signs of disease recurrence^9^. Therefore, it is difficult to compare these results with the current study.

Seo et al. reported that the cumulative recurrence rate of ovarian endometrioma was 10.2% at 60 months in patients 40–45 years of age. This study also reported no differences in recurrence with or without post-operative medication^10^, which is consistent with our findings. However, Seo et al. only compared 61 patients, divided into a no medication group (n = 40) and a gonadotropin-releasing hormone (GnRH) agonist + oral contraceptives (OC) group (n = 21)^10^. Thus, this study may also not be directly comparable to the current report.

In addition to the above mentioned studies, other reports including randomized controlled trials have demonstrated that post-operative medical treatment markedly reduces the recurrence rate of endometrioma^12–17^. Therefore, long-term medical treatment to prevent recurrence is routinely recommended^8^. Post-operative medical treatments including OC, GnRH agonists, and progesterone, commonly used to suppress possible residual lesions due to the estrogen-reducing effects^3,18^. However, each of these treatments has reported adverse effects. The GnRH agonist affects bone mineral density (BMD); in previous studies, adults lost 5–8% of spine BMD after only 3–6 months of GnRH agonist treatment^19–21^, and decreased BMD may not return to baseline after cessation of treatment^22,23^. Therefore, the GnRH agonist is typically combined with add-back therapy and only used as a short-term treatment. Progestin has also been shown to decrease BMD with long-term use. Momoeda et al*.* reported a 1.6 ± 2.4% and 1.7 ± 2.2% decrease in lumbar BMD at 24 and 52 weeks of dienogest use^24^. Seo et al. compared OC after 6 months of GnRH agonist with add-back therapy versus dienogest for 24 months and showed that lumbar BMD significantly decreased after the first 6 months in GnRH agonist + OC (3.5%) and dienogest (2.3%), and that there were no differences between the two treatments^25^. In the GnRH agonist + OC group, BMD increased with time after starting OC, and in the dienogest group, BMD did not decrease further after the first 6 months; therefore, there was no further decease in BMD until 24 months in both groups^25^. In cases of OC, even in healthy women, the U.S. Medical Eligibility Criteria for contraceptive use categorized OC as category 2, indicating that the theoretical or proven risks of OC must be considered^26^. Oral contraceptive is usually contraindicated in women with conditions such as hypertension, cardiovascular disease, deep vein thrombosis, diabetes mellitus accompanied by organ failure, gallbladder disease, and other malignant diseases such as breast cancer^26^, and these diseases are typically increased in older women. Coronary heart disease prevalence in women aged 20–39 years is less than 1%, but after age 40, the percentage increases to greater than 5%^27^. In clinical practice, patients between ages of 40 and 49 years of age are limited by cardiovascular disease and other conditions when choosing hormonal therapy.

However, the efficacy or necessity of post-operative medication was not extensively investigated in women aged 40 years or higher. Considering the limited previous reports on ovarian endometrioma in women aged 40–49 years, our study provides interesting and novel data for the post-operative management of this patient group.

A major strength of our study is the inclusion of a large cohort of patients. However, this study has several limitations. Due to the retrospective study design, some potential selection biases for post-operative medical treatment may have occurred. The decision of post-operative medical treatment was selected according to the surgeon recommendation and each patient’s preferences. Yet, additional biases were minimized by excluding pregnancy, menopause, and oophorectomy cases, which are known to reduce disease recurrence. Second, all included patients were from a single institution. However, we analyzed a larger number of patients compared to previous studies. Third, disease recurrence was defined on the basis of ultrasound findings and did not reflect pain levels. It is difficult to discriminate the pain caused by other gynecologic diseases, such as myoma or adenomyoma, which were present in 329 (80.6%) of included patients in this study. Therefore, in some cases, we considered the time interval from the start of post-operative medical treatment to when the patients complained of pain after surgery, for cases where less than 2 cm of ovarian endometrioma was indicated by ultrasound. These cases were not included as true recurrence. Fourth, we did not compare types of post-operative medical treatment: GnRH agonist, progestin, OC, or levonorgestrel-releasing intrauterine device. Since each patient was treated on a case-by-case basis, the medical treatments were not consistent among cases; therefore, this comparison was difficult in our study group. To overcome these limitations, future large-scale, prospective, observational or comparative studies are required.

In conclusion, the relatively low cumulative recurrence rate of ovarian endometrioma seemed to be in women aged 40–49 years, regardless of post-operative medication use after surgery, and the only risk factor for recurrent ovarian endometrioma was large cyst size (cyst size > 5.5 cm). In this study, we did not confirm the risk reducing effect of recurrence in post-operative medication use, but more research is needed to determine whether post-operative medication use really does not further decrease risk in this age group.

Due to the possible risks and benefits of hormonal treatment, it is possible to omit post-operative medical treatment for the prevention of disease recurrence in this age group with informed consent. However, more research is needed to determine whether post-operative medication use decreases risk of recurrent in this age group.

## Data Availability

Data will be available upon reasonable request from the corresponding author. However, the data cannot be made public to maintain women’s privacy and legal reasons as it contains private health information along with identifiers.
